# Various Surgical Interventions in Treating Odontogenic Keratocyst: A Radiological Case Report

**DOI:** 10.3390/healthcare11030416

**Published:** 2023-02-01

**Authors:** Gabriela Luminița Gelețu, Alexandru Burlacu, Elena-Raluca Baciu, Diana Diaconu-Popa, Alice Murariu, Liliana Georgeta Foia, Loredana Ungureanu, Neculai Onică

**Affiliations:** 1 Department of Surgery, Faculty of Dental Medicine, University of Medicine and Pharmacy, “Grigore T. Popa”, 700115 Iasi, Romania; 2 Department of Internal Medicine, Nephrology, Geriatrics, Faculty of Medicine, University of Medicine and Pharmacy, “Grigore T. Popa”, 700115 Iasi, Romania; 3 Department of Implantology, Removable Dentures, Dental Technology, Faculty of Dental Medicine, University of Medicine and Pharmacy, “Grigore T. Popa”, 700115 Iasi, Romania; 4 Department of Morphopathology, Faculty of Medicine, University of Medicine and Pharmacy, “Grigore T. Popa”, 700115 Iasi, Romania; 5 Specialist Oral and Maxillofacial Surgery, Private Practice, 700377 Iasi, Romania

**Keywords:** odontogenic keratocyst, decompression, marsupialization, recurrence, orthopantomography, cone-beam computed tomography, histopathology

## Abstract

The odontogenic keratocyst (OKC) is among the most aggressive odontogenic cysts because of its high recurrent rate. This study’s objective was to describe a 7-year radiological and clinical follow-up of an OKC with two recurrences and a combination of surgical treatments. The cyst contents were drained at the biopsy to allow decompression, and then marsupialization (Partsch I) was carried out with good results. In the following two years, the endodontic and radiological evaluation of the neighboring teeth indicated endodontic avoidance. The remaining OKC enucleation (Partsch II) with chemical curettage and suture was performed two years after the first presentation, and the multifocal recurrences developed were also treated by enucleation and chemical curettage. At the last radiological follow-up, no recurrence was evident. Based on our experience, we concluded that patients diagnosed with a OKC should be radiologically evaluated at least once a year for at least 7 years, the endodontic diagnosis and treatment of neighboring teeth should be performed as early as possible, and the combination of surgical treatment and the long follow-up period is challenging.

## 1. Introduction

The odontogenic keratocyst (OKC) is particularly interesting because of its aggressive nature, very high recurrence rate, and disputed treatment modalities. The term “odontogenic keratocyst” was first reported by Phillipsen [1] as a separate clinical entity in 1956. The cyst is considered to derive from the cell remnants of the dental lamina. In 1992, the World Health Organization (WHO) introduced the term “Odontogenic Keratocyst”, synonymous with “primordial cyst”, to denote benign cysts of odontogenic origin and specific histological appearance [2]. However, in 2005, the WHO reclassified this pathology as a benign keratocystic odontogenic tumor (KCOT) because of its high risk of recurrence, aggressive behavior, the occurrence of satellite cysts, mutations of the tumor-suppressor gene (PTCH1), and the association with Gorlin–Goltz syndrome. According to the WHO’s last classification (2017), the keratocyst is an odontogenic cyst lined by “parakeratinized stratified squamous epithelium with palisading hyperchromatic cells” [3].

The OKC usually appears in the second and third decades of life with male predilection and grows within the medullary bone without significant cortical modifications. It can be located anywhere in the jaw, rarely in the maxillary sinus, but it is commonly seen in the posterior part of the mandible. In many cases, patients are free from symptoms until the cyst reaches a large size and involves the entire ascendant ramus and the mandibular body [4,5]. 

Radiographically, it presents as a unilocular or a multilocular radiolucency and may be confused with ameloblastoma or dentigerous cyst [6]. To establish an accurate final diagnosis of an OKC, a detailed intra- and extraoral clinical evaluation, thorough radiographic analysis, and histopathological examination (the gold-standard investigation) are required, particularly the latter.

The choice of treatment is based on the size of the cyst, the recurrence status risk, and the radiographic evidence of cortical perforation. Therapeutic interventions of OKCs include marsupialization, decompression, enucleation, enucleation with chemical curettage (Carnoy’s solution), enucleation with liquid nitrogen (cryodestruction), peripheral ostectomy, and jaw resection [7,8]. 

Enucleation with chemical curettage using Carnoy’s solution (CS) application has been proposed as a therapeutic strategy with a lower recurrence rate than enucleation alone. Described in 1933, Carnoy’s solution (CS) contained 1 g of ferric chloride (FeCl_3_) dissolved in 6 mL of pure alcohol, 3 mL of chloroform, and 1 mL of glacial acetic acid. According to certain studies, Carnoy’s solution contributed to favorable results in terms of morbidity and lesion recurrence rate [9,10]. Based on research on animals, in 2013, the Food and Drug Administration (FDA) prohibited CS because it included chloroform, which was classified as “reasonably anticipated to be a human carcinogen” [11]. To avoid the risk of carcinogenicity, the surgeons started to use the modified Carnoy solution, which does not contain chloroform or 5-fluorouracil (5-FU).

The appropriate treatment for OKCs is still controversial [12], with recurrence rates ranging from less than 10% to more than 60% [13,14].

There are different recurrence theories of the OKC, including the incomplete removal of the original cyst lining, the growth of a new OKC from small satellite cysts, or odontogenic epithelial rests left behind by surgery or the development of an unrelated OKC in an adjacent region of the jaws [15]. Keratocysts enucleated in one piece recurred significantly less often than cysts enucleated in several pieces [16].

The recurrence rate of keratocysts associated with infection, fistula, or the perforation of the bony wall is higher than that of OKCs without these features [17]. Recurrence is also found more frequently in cysts with multilocular radiographic appearance than in unilocular cysts. The type of surgery may not be the only factor for relapse, and some authors suggested that recurrence may be related to the biological nature of the lesion itself and the expression of proliferative markers [18,19].

**Figure 1:**
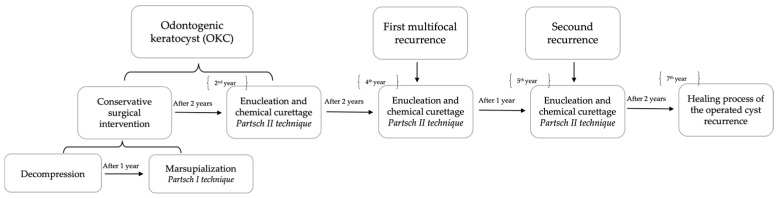
The patient evolution scheme from the first presentation.

This paper aimed to present a 7-year follow-up of an OKC in a 32-year-old patient who developed two recurrences and a combination of surgical treatments (Figure 1). The first surgery considerably reduces the cyst’s size; and, in the second, enucleation and chemical curettage eliminate the cyst lining. The first recurrence was multifocal, with one at the anterior part of the right ascended ramus, and the other at the right mandibular body in the apical region of the first and second mandibular molars. The second recurrence was in the same place but at the right mandibular body, at the apical region of the first nontreated molar.

## 2. Case Presentation

In 2015, the dentist referred a 32-year-old man to maxillofacial surgeons for continuous mild gnawing pain on the right part of the mandible for the past four weeks, as well as signs of inferior alveolar nerve paresthesia. He had no medical or surgical history and was a lifelong non-smoker with no significant family history. Right-face asymmetry was observed on extraoral examination; however, there were no skin changes or submandibular lymphadenopathies.

The intraoral examination showed a buccal cortical bulging in the posterior right mandibular region, while the cortical bone at the molar #46 apical levels was soft in consistency and fluctuant. The vitality tests of the teeth #46, #47, and #48 were positive.

**Figure 2:**
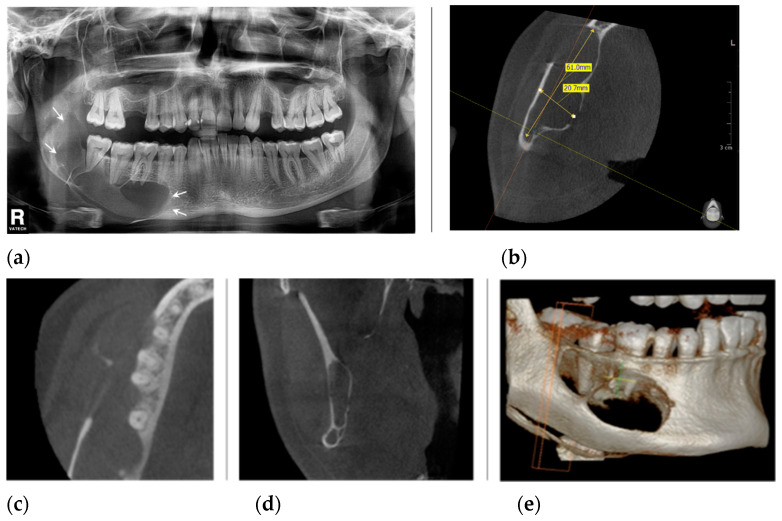
The radiological aspect before surgical treatments: (**a**) panoramic radiograph shows a large multilocular lesion occupying the body of the mandible, from the right second premolar to the ascendent ramus (arrows), without involving the condyle or coronoid process, and inferior displacement of the mandibular canal; (**b**) the CBCT, axial view shows the lesion measuring 61 mm in length and 20.7 mm in width; (**c**) the CBCT image indicates that the apices of the first and second molar are in the unaffected bone structure; (**d**) the CBCT, coronal view of the ascending ramus, indicates the condyle is not involve; (**e**) the CBCT, 3D reconstruction view.

Orthopantomography (Figure 2a), supported by cone-beam computed tomography (CBCT) images (Figure 2b–e), showed a large well-defined multilocular radiolucency bounded by corticated margins, with few and incomplete septa within the bone lesion, and inferior displacement of the mandibular canal. Teeth #46, #47, and #48 remained within the cystic lumen without displacement or root resorption (Figure 2c). Figure 2d shows that the condyle was unaffected.

We planned to do a biopsy and a two-stage conservative surgery (decompression at the first stage and marsupialization at the second stage) to reduce the cyst’s volume, and than a radical surgery enucleation and chemical curettage (Partsch II technique).

After the patient was informed of the diagnosis, the prognosis with and without treatments, the therapeutic steps, benefits, procedure risks, and probable adverse reactions, informed consent was signed.

The extraction of tooth #48 under local anesthesia was performed, and the aspirate of cystic content carried out through the alveolus had a creamy appearance. The biopsy was harvested after the margins of the cortical bone were removed. The cavity was irrigated through the empty socket with abundant normal saline and a 5% povidone-iodine solution. For decompression, a plastic drain tube was introduced into the cystic cavity and sutured at the socket margins, which permits the irrigation of the cystic cavity.

**Figure 3:**
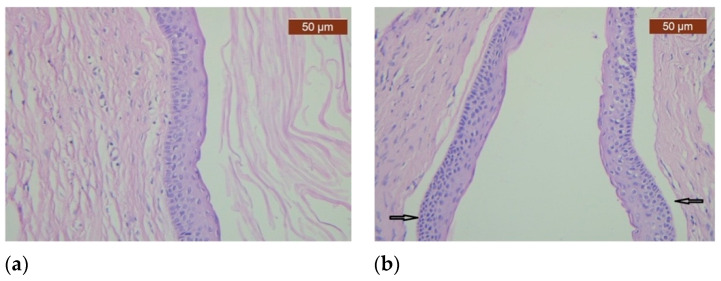
Histopathological examination of the specimen shows a cystic space delineated by 6–8-layers-thick squamous epithelium with basal layer arranged in palisaded manner. (**a**) The cyst wall’s photomicrograph shows that the cyst’s inner surface is lined by squamous epithelium with orthokeratinization; (**b**) The epithelium and connective tissue are focally detached (arrows).

The histopathological diagnosis revealed a cystic structure lined on the inner part via squamous epithelium with focal parakeratinization, the basal layer with palisade-arranged hyperchromic nuclei, and the rectilinear relief. Inside the cyst, we noticed orthokeratotic keratin lamellae. Subepithelial connective tissue had fibrosis and edema with a tendency to form cleavage spaces without signs of inflammation (Figure 3a,b).

A general clinical examination including Dermatological Department visit, blood tests, and chest and skull X-rays ruled out in the Gorlin–Goltz syndrome (GGS). To establish a diagnosis of GGS, at least two major and one minor or one major and three minor clinical and radiological criteria must be present, and none were present in our patient. 

For one year, the patient irrigated the cystic cavity with normal saline every day. Every month, as we decided at the initial management, we verified the permeability and, if necessary, resutured the tube at the neighboring teeth (#47, #46). At each follow-up, the first tube was replaced with a shorter one. In the same time, an endodontic evaluation with positive vitality response was performed twice at the dentist, and the decision to avoid endodontic treatment was chosen.

**Figure 4:**
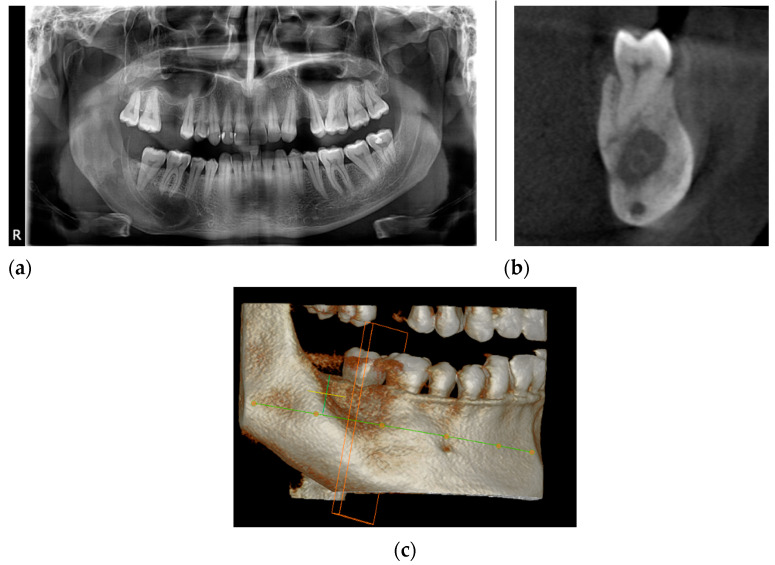
The radiological aspect after one year of decompression: (**a**) panoramic radiography shows a reduction in the size of the cyst; (**b**) the CBCT, coronal view indicates the position of the tube in the cyst cavity; (**c**) the CBCT, 3D reconstruction view.

A year after the decompression, the patient returned (Figure 4a–c) for the pre-planned marsupialization, using a Partsch I technique (transforming the cyst into an open pouch, allowing continuous drainage).

Patient consent was taken, and local anesthesia and extended incision from the second molar up to the anterior part of the ramus were performed. The bony margins forming the opening for the cystic cavity, along with its attached overlying mucosa, were removed. After the cavity irrigation, the cystic lining (which was much ticker than the oral mucosa) was folded, and the edges were sutured with a mucosal flap using a 6.0 Vicryl. The 5% povidone-iodine-soaked gauze was packed in the cystic cavity. For one year, the gauze was replaced every two weeks, taking care to ensure that it did not come into the mouth. At each follow-up appointment, we gradually reduced the size of the gauze. In our case, no antibioterapy was prescribed; oral hygiene was enhanced with 2% chlorhexidine for the first four weeks after surgery, and the diet was normal.

**Figure 5:**
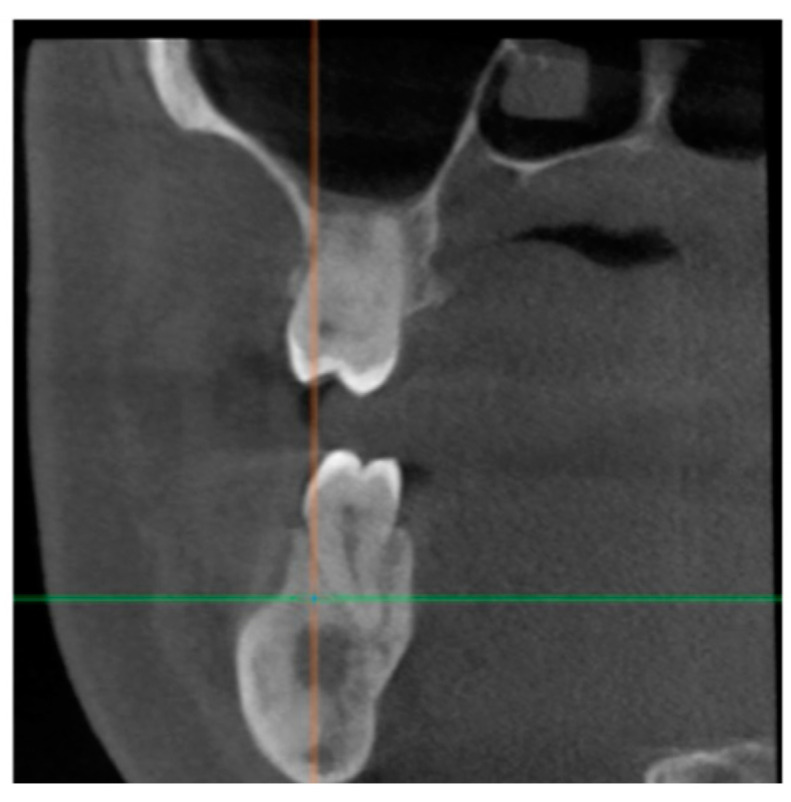
CBCT image of the bone healing process in the tooth #47 periapical area.

The decision to forego endodontic treatment for molar #47 was made by the endodontic specialist based on CBCT image (Figure 5) analysis and a positive vitality test.

**Figure 6:**
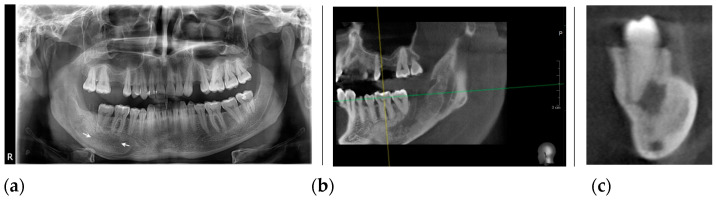
The radiological aspect: (**a**) Orthopantomography shows the healing process in the ascendant ramus, and the remaining osteolytic lesion at the apical region of the first and second mandibular molars (arrows), one year after marsupialization; (**b**) The CBCT, sagittal view indicates unmodified bone structure near the molar #46 apices; (**c**) The CBCT, sagittal view of molar #47 demonstrates the lesion contact and the enlarger of periodontal space.

A year from the marsupialization (and two years after the first presentation), the radiological aspect showed complete healing in the posterior part of a former lesion at the level of the ascendent ramus. However, it remained the same in the anterior part, at the level of the mandibular body, under the first and second mandibular molars roots (Figure 6a,b). The conservative advantage of the decompression and marsupialization was demonstrated in this case; the reduced lesion volume made the second stage of surgery more comfortable for the patient and the surgeon by reducing the risk of inferior alveolar nerve (IAN) damage. No paresthesia of IAN was found two years after decompression.

The surgical treatment proposed for the remaining 2 cm OKC at the apical level of teeth #46 and #47 was enucleation and chemical curettage (Partsch II technique) associated with the second molar extraction (Figure 6c). Chemical curettage consisted of applying modified Carnoy’s solution into the cyst cavity. The solution induced superficial tissue necrosis and helped to eliminate the tumor remnants. After informed consent and local anesthesia, more lining cyst pieces and enucleation were performed with the extraction of the second mandibular molar in the same session. Modified Carnoy’s solution was applied to the profound cavity walls for 3 min. 

The endodontist decided against performing endodontic therapy on molar #46 based on radiological images and a positive vitality test.

The histological assessment confirmed parakeratinized squamous epithelium with a prominent palisade-arranged basal layer; the finding confirmed the presence of an OKC.

**Figure 7:**
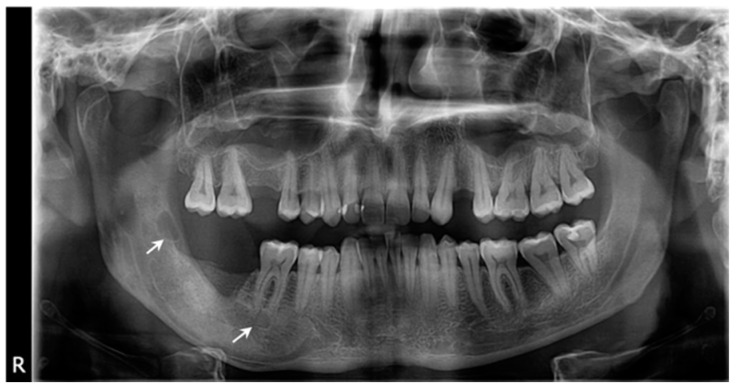
The initial aspect of two unilocular radiolucencies: one at the anterior part of the ascendent ramus (arrow), and the anterior one in the apical root of tooth #46 (arrow), without clear delimitation.

At the clinical and radiological follow-up, nine months after the enucleation and chemical curettage, two unilocular radiolucencies (one at the ascendent ramus and another under the first molar) were visible on the orthopantomography (Figure 7). 

**Figure 8:**
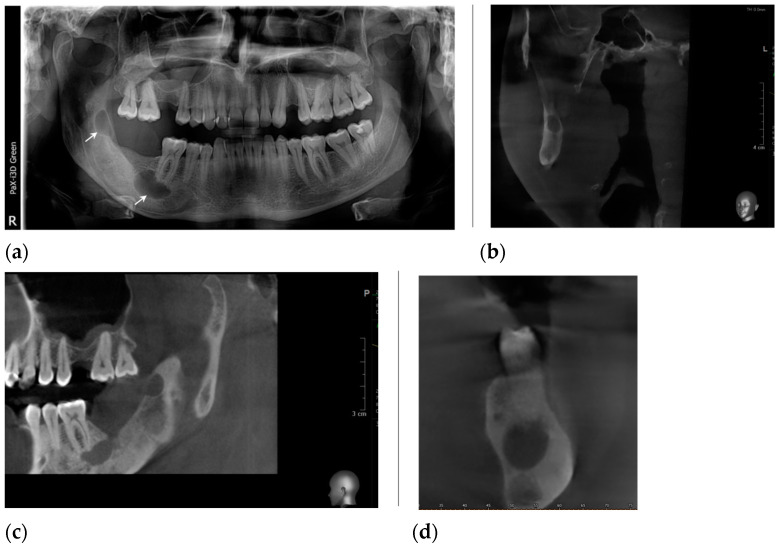
The radiological aspects: (**a**) panoramic radiograph demonstrate two well-defined osteolytic lesions: one at the apical region of tooth #46 (arrow), without a mandibular canal involved, and another at the anterior margin of the ascendant ramus (arrow); (**b**) the CBCT, coronal view of the affected ascending ramus; (**c**) the CBCT, coronal view highlights the panoramic radiograph findings; (**d**) the CBCT, sagittal view demonstrates the lesion contact with the molar #46.

The patient returned very late because of personal motivations in the COVID pan-demic period (after another 15 months from the diagnosis of the recurrence) and the radi-ological images demonstrated two separated radiolucent unilocular lesions (Figure 8a,b) at both margins of the previously operated OKC. The diagnosis was multifocal OKC re-currence. Because of the strong relationship between the anterior lesion and the apices of tooth #46 (Figure 8c,d), an endodontic treatment was chosen.

The multifocal recurrent surgical treatment was decided, and informed consent was obtained. Due to the failure of molar #46 endodontic treatments, the decision to extract it was taken, and an apicectomy was not taken into account because of the high aggressivity of this type of cyst to prevent a recurrence. The surgery was completed under local anesthesia. It consisted of enucleation of both cystic lesions and chemical curettage in the same procedure associated with tooth #46 extraction. Biopsy revealed parakeratinized odontogenic keratocyst in both lesions.

**Figure 9:**
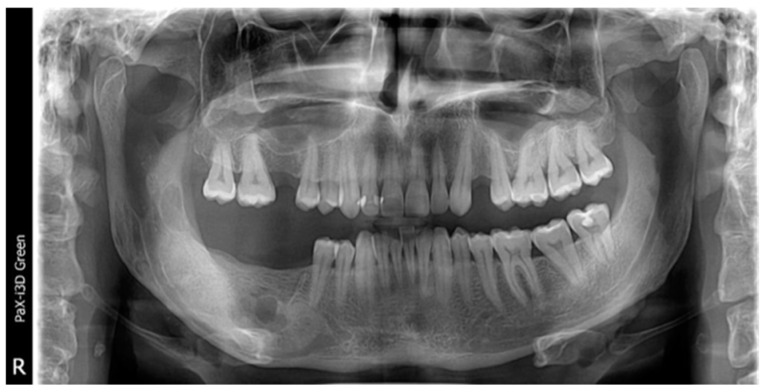
Orthopantomography, a year from recurrence surgery, revealed a bone healing process.

One year after the enucleation of multifocal OKC recurrence, at the clinical and radiological control, the aspect of the bone demonstrated a process of normal healing (Figure 9).

**Figure 10:**
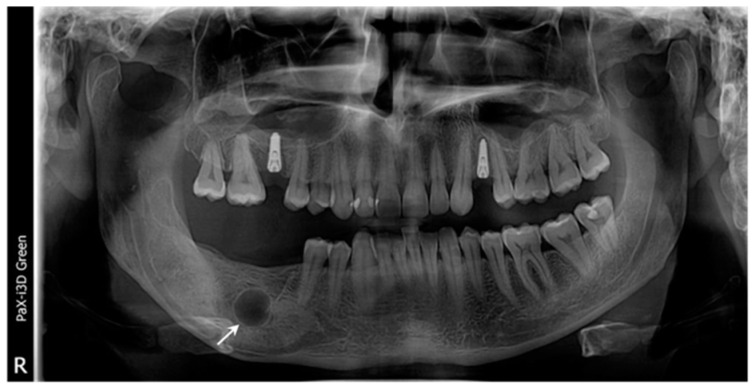
Orthopantomogram shows the second recurrence after one year, a unilocular radiolucency lesion, bounded by corticated well-defined margins (arrow), in the same place as the first operated recurrence.

A year later, at the subsequent follow-up, a new OKC recurrence (the second one) appeared at the radiologic control (Figure 10), situated at the anterior aspect of the previously operated cyst recurrence (the second recurrence in the same place). The decision to enucleation was taken, and informed consent was obtained. The surgery consisted of enucleation in one piece with chemical curettage and suture.

**Figure 11:**
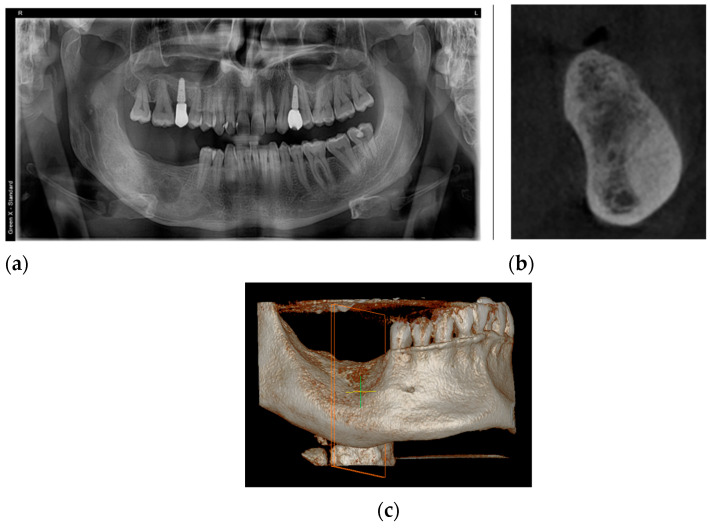
The aspect of the healing process, two years after recurrence surgery: (**a**) orthopantomography image; (**b**) cone-beam computerized tomography image; (**c**) the CBCT, 3D reconstruction view.

At the last follow-up, two years after enucleation, orthopantomography (Figure 11a), confirmed by cone-beam computerized tomography (Figure 11b,c), revealed a regular aspect of the healing process of the operated OKC recurrence. 

The patient did not develop any recurrence despite the presence of focal parakeratinized area and cleavage spaces histologically. Seven years after the first presentation, the patient had no facial asymmetry and no IAN paresthesia and was proposed for dental implants at the right mandibular ramus. 

## 3. Discussion

Despite extensive research, there is no consensus regarding the aggressive behavior of the OKC and the cause of recurrences. The recurrences might be explained by different causes, such as the incomplete removal of the highly active basal layer of the epithelial cyst lining, the growth of a small intramedullary satellite cyst left behind by conservative treatment, and the development of lesions localized in the adjacent region of the jaws [20,21]. For our case, the radiological and pathological results, concluded for OKC. Gorlin–Goltz syndrome was excluded.

To some authors, marsupialization has a high success rate, and the OKC treatment protocol based on decompression offers a conservative and effective option with low morbidity [22]. We chose this technique to reduce aggressive behavior based on the osmotic tension exerted on the adjacent tissues. The two-stage treatment ensures complete removal without causing injury to the surrounding structures, and marsupialization was found to have a lower recurrence rate [23].

OKC has been subject to a lot of debates and controversies because of its diverse nature and high recurrence rates [8].

Blanas et al., in a systematic review, found a recurrence rate of approximately 17% to 56% when the OKC was treated by simple enucleation, which explains the addition of Carnoy’s in the cystic cavity for 3 min after enucleation, thus reducing the recurrence rate to 1.6%, i.e., a rate comparable to the resection one but without associated morbidity [24]. 

According to Dashow et al. [25], the simple enucleation and curettage with MC results in a high recurrence rate of 35%. The MC has shown to be far less effective than the original compound (EC). In 2021, a retrospective cohort study showed that chemical curettage in the form of MC as an adjuvant to EC and peripheral ostectomy remains among the most successful treatments for OKCs [11]. Concerning our case, we could not establish that enucleation and chemical curettages with MC influenced the recurrence rate.

The young patient had a sizeable multiloculated cyst and a period of the volume-reducing lesion by decompression and marsupialization of two years with well-demonstrated advantages. Marsupialization permits a more gradual decompression of the cyst cavity, which, in our perspective, after the first year, remains too large for ambulatory surgery.

Despite a rigorous enucleation treatment and chemical curettage, two recurrences appeared in the same place of the mandibular body. The recurrences could appear as a result of the posterior location of the lesion with a problematic approach, extensive and multilocular lesions that could not be resected easily, the presence of daughter cysts, and because the tumor could have a cleavage space between the epithelium and connective tissue that made the cyst to be fragile and friable (leaving epithelial remnants in place that could be at the origin of relapses) [6,26]. Despite the teeth #46 and #47 positive vitality responses, endodontic treatment should be performed. The lack of endodontic treatment, or the apicectomies of tooth #46, could also lead to recurrence. More attention has to be given to early endodontic diagnosis and treatment of teeth #46 and #47, that conclude the importance of early neighbored teeth clinical and radiological evaluation in OKC treatment. 

Some histopathological aspects are risk factors for recurrence, such as parakeratinization, budding, daughter cysts [27], and the tendency to cleave between the epithelium and connective tissue due to the fragility of the epithelial lining [28]. These aspects, together with insufficient enucleation, could cause aggressive behavior. In our case, the last surgery specimen also revealed orthokeratinized epithelial lining, a sign that could be associated with a better prognosis, according to the literature [29]. 

After an extended (7-year) follow-up, with two recurrences, a multifocal one and a new OKC recurrence, the final result was acceptable, with an appropriate intraosseous bone structure at surgical sites and no aesthetic compromise. The patient is still in the follow-up period.

In addition, our case exemplified the value of multidisciplinary patient management, including dermatology, endodontics, radiology, maxillofacial surgery, and pathology.

## 4. Conclusions

Based on our case, both conservative procedures worked synergistically and, instead of a two-year decompression, we opted to undergo the marsupialization and to avoid a extensive surgery. The decompression and marsupialization decreased the lesion volume and allowed for a second stage of surgery to reduce morbidity and improve the patient’s postoperative quality of life. Histopathological aspects such as parakeratinization, cleavage space, and daughter cysts are essential in diagnosis and prognosis risk evaluation. The endodontic diagnosis based on radiological images must be properly performed and as soon as possible.

Conservative surgical therapy to limit morbidity, the endodontic treatment of adjacent teeth to minimize the risk of recurrence, and the long-term follow-ups with periodic radiographic examinations are essential.

Protocols for the early diagnosis of OKCs should be established to prevent radical treatment with a large loss of substance. 

## Data Availability

Not applicable.
